# Toxic Oligomeric Alpha-Synuclein Variants Present in Human Parkinson’s Disease Brains Are Differentially Generated in Mammalian Cell Models

**DOI:** 10.3390/biom5031634

**Published:** 2015-07-22

**Authors:** Wei Xin, Sharareh Emadi, Stephanie Williams, Qiang Liu, Philip Schulz, Ping He, Now Bahar Alam, Jie Wu, Michael R. Sierks

**Affiliations:** 1 Chemical Engineering, Arizona State University, Tempe, AZ 85287-6106, USA; E-Mails: wie.xin@asu.edu (W.X.); sharareh.emadi@asu.edu (S.E.); swilli4@asu.edu (S.W.); Philip.Schulz@asu.edu (P.S.); pinghe2@asu.edu (P.H.); nalam1@asu.edu (N.B.A.); 2 Division of Neurology, Barrow Neurological Institute, Phoenix, AZ 85013, USA; E-Mails: qiang.liu@asu.edu (Q.L.); jie.wu@DignityHealth.org (J.W.)

**Keywords:** Parkinson’s disease, α-synuclein, aggregation, scFv antibody, neuroblastoma cells (SH-SY5Y)

## Abstract

Misfolding and aggregation of α-synuclein into toxic soluble oligomeric α-synuclein aggregates has been strongly correlated with the pathogenesis of Parkinson’s disease (PD). Here, we show that two different morphologically distinct oligomeric α-synuclein aggregates are present in human post-mortem PD brain tissue and are responsible for the bulk of α-synuclein induced toxicity in brain homogenates from PD samples. Two antibody fragments that selectively bind the different oligomeric α-synuclein variants block this α-synuclein induced toxicity and are useful tools to probe how various cell models replicate the α-synuclein aggregation pattern of human PD brain. Using these reagents, we show that mammalian cell type strongly influences α-synuclein aggregation, where neuronal cells best replicate the PD brain α-synuclein aggregation profile. Overexpression of α-synuclein in the different cell lines increased protein aggregation but did not alter the morphology of the oligomeric aggregates generated. Differentiation of the neuronal cells into a cholinergic-like or dopaminergic-like phenotype increased the levels of oligomeric α-synuclein where the aggregates were localized in cell neurites and cell bodies.

## 1. Introduction

Parkinson’s disease (PD) is a progressive neurodegenerative disorder that affects approximately 1% of people aged 65 and older [1]. Clinically, PD is characterized by severe motor dysfunction including muscular rigidity, uncontrollable resting tremor and bradykinesia [1,2]. Pathologically, PD is characterized by the selective and progressive loss of dopaminergic neurons in the substantia nigra pars compacta, resulting in reduction of dopamine in its striatal projections and other brainstem regions. PD is also characterized by the presence of cytoplasmic and neuritic fibrillar inclusions known as Lewy bodies (LB) and Lewy neurites in the surviving dopaminergic neurons and other affected areas of the central nervous system [3,4]. The presence of LBs is related with either neuronal dysfunction or neuronal death depending on the brain region and the stage of disease [5,6]. The protein α-synuclein was shown to be a primary constituent of LBs in sporadic PD and other neurodegenerative diseases [7,8,9,10,11]. While most cases of PD are sporadic, both genetic mutations in the α-synuclein gene (A53T, A30P, and E46K) and gene duplication resulting in overexpression of α-synuclein have been associated with familial forms of PD [2,12,13]. A small natively unstructured protein (14 kDa), α-synuclein is expressed mainly in brain tissues and localized in the presynaptic terminals of neurons [14]. α-Synuclein can misfold and assemble *in vitro* into a variety of β-sheet based aggregates including small soluble oligomeric, larger soluble protofibrillar, and fibrillar species. Environmental factors can influence α-synuclein folding as metal ions including aluminum, copper, iron, and calcium, heparin, catecholamines such as dopamine, and pesticides such as rotenone have all been observed to facilitate stabilization of α-synuclein into its β-sheet conformation [15,16,17,18,19]. While several morphologies of α-synuclein can be generated *in vitro*, increasing evidence suggest that different soluble oligomeric species rather than mature fibril forms are responsible for neuronal dysfunction and toxicity in PD disease [20,21,22,23,24]. Aggregated forms of α-synuclein have been shown to induce toxicity in dopaminergic neurons *in vivo* [25], and different toxic mechanisms have been associated with various different aggregated morphologies [26]. Elevated extracellular levels of oligomeric α-synuclein have also been detected in blood plasma and cerebrospinal fluid in PD patients, implicating these aggregates as important in the etiology of PD [27,28,29,30,31].

While α-synuclein is considered an important target for studying PD, its role in the progression of PD pathogenesis is still largely unknown because of the complex array of different α-synuclein morphologies that exist and the lack of suitably selective tools and reagents to probe the roles of these different species in PD models and tissues. In our lab, we have generated reagents that recognize several distinct morphologies of α-synuclein including two different oligomeric forms: the D5 antibody fragment binds to an *in vitro* generated SDS-stable dimeric and tetrameric α-synuclein, and the 10H antibody fragment binds to an *in vitro* generated SDS-stable trimeric and hexameric α-synuclein [20,21,32]. Here, we utilized the two different α-synuclein oligomer specific antibody fragments (D5 and 10H) [20,21] to identify the predominant cytotoxic species present in brain homogenates from post-mortem human PD brain tissue. The PD brain homogenates were shown to be substantially more cytotoxic to SH-SY5Y cells compared to brain homogenates from age matched cognitively normal brain homogenates. The increased cytotoxicity could be largely blocked in a concentration dependent manner by addition of D5 and/or 10H, indicating that most of the increased neuronal toxicity in human PD brain tissue samples compared to age matched control samples is attributable to the presence of specific oligomeric α-synuclein species. We then studied how well mammalian cell lines replicate the presence of these toxic α-synuclein species. We probed for the presence of both the D5 and 10H reactive toxic α-synuclein aggregates in different mammalian cell lines expressing endogenous levels of α-synuclein including non-differentiated and differentiated human neuroblastoma cells (SH-SY5Y), Chinese hamster ovary (CHO) cells and human embryonic kidney (HEK) cells. We also utilized HEK, CHO and SH-SY5Y cells that overexpress α-synuclein to determine whether overexpression of α-synuclein can alter the protein aggregation pathway. Undifferentiated SH-SY5Y cells have been widely used as a PD cell model; however, this cell line can be differentiated to cholinergic-, adrenergic-, or dopaminergic- phenotypes by altering growth conditions. Sequential exposure of SH-SY5Y cells to retinoic acid and brain derived neurotrophic factor (BDNF) in serum-free medium yields homogeneous populations of fully differentiated cholinergic-like and dopaminergic-like neuronal cells which are very comparable to primary neurons [33,34,35,36]. Here, we show that the choice of cell model and differentiation state can quite dramatically impact the α-synuclein aggregation process.

## 2. Results and Discussion

The protein α-synuclein has been strongly linked with PD and other related neurodegenerative disorders [7,10,37]. The α-synuclein protein occurs *in vivo* in various forms and morphologies [8,38,39], and can interact with membranes [40,41,42], other proteins such as tau, p25alpha, tubulin, and transcription factor ELK-1 [43,44,45], metal ions including aluminum, copper, calcium, and iron [19], and catecholamines such as dopamine [15,46]. The various interactions can facilitate formation of different aggregate α-synuclein species. While ample evidence indicates that α-synuclein plays an important role in the pathogenesis of PD, the impact of the various conformations of α-synuclein in the progression of PD is much debated and poorly understood. Elevated levels of oligomeric α-synuclein in CSF and plasma from PD patients and in the brain samples of Lewy bodies from dementia patients has been demonstrated [28,47]. We developed antibody fragments that selectively recognize two different toxic oligomeric α-synuclein species, D5 recognizing an *in vitro* generated SDS stable dimeric species [20], and 10H recognizing an *in vitro* generated SDS trimeric species [21]. We showed that both of the oligomeric aggregate species occurred in human post-mortem PD brain tissue [20,21] and CSF samples [30]. Here, we show that the D5 and 10H reactive oligomeric α-synuclein variants account for the bulk of cytotoxicity induced by human PD brain tissue homogenates.

**Figure 1:**
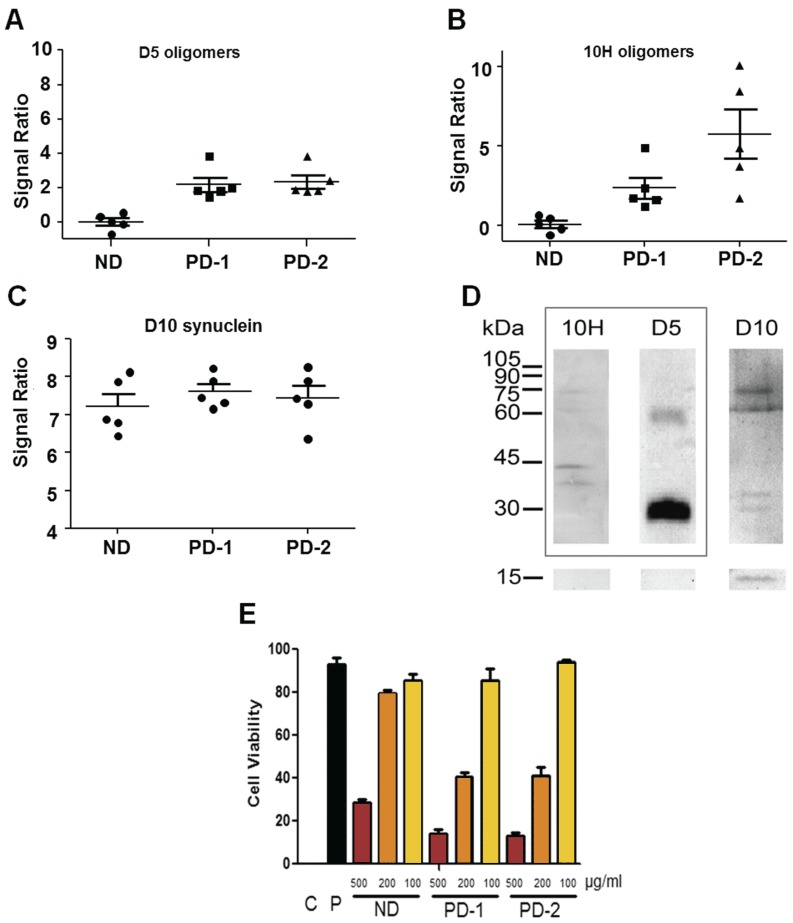
D5 and 10H reactive α-synuclein oligomers in human patient brain samples and their cytotoxicity in SH-SY5Y cell model. (**A**)–(**C**) Relative amounts of D5 and 10H reactive α-synuclein oligomers and D10 α-synuclein (reacting with total α-synuclein) present in different human PD and control brain homogenates; (D) Western blot of D5, 10H and D10 scFv binding specificity with α-synuclein aggregates (adapted from [20,21]); (**E**) Toxicity of homogenized brain tissue from PD-1, PD-2 and ND control brain pools towards SH-SY5Y cells. C is a SHSY5Y cell-free control corresponding to 0% cell viability limit. P is a healthy SHSY5Y cell control corresponding to 100% cell viability limit.

We utilized the human neuroblastoma cell line, SH-SY5Y, to determine cytotoxicity of homogenized post-mortem human PD and control brain tissue samples *in vitro* [48]. We first quantified the levels of both D5 and 10H reactive α-synuclein aggregates in the human PD and control patient brain homogenates using capture ELISA [49] (Figure 1). The PD brain homogenates showed significantly higher levels of oligomeric α-synuclein than the age-matched control samples, though the distribution of D5 and 10H oligomeric species varied among PD patients. We separated the 10 different PD brain homogenate samples into two different PD mixes; PD-1 containing five tissue samples that have similar levels of both D5 and 10H reactive oligomeric α-synuclein (Figure 1A), and PD-2 containing tissue samples that have substantially higher levels of 10H reactive oligomeric α-synuclein compared to D5 reactive α-synuclein levels (Figure 1B). Total α-synuclein levels in the control, PD-1 and PD-2 samples were all similar (Figure 1C). The different binding specificities of the D5, 10H and D10 scFvs toward different aggregate morphologies of α-synuclein are shown in Figure 1D.

After identifying the concentration of the different oligomeric α-synuclein species in the brain homogenate samples, we then determined the toxicity of the samples when incubated with SH-SY5Y cells. We varied the concentration of brain tissue homogenate (1000, 500, 200, 100, 50, 20 and 0 µg/mL total protein) incubated with the cells to determine toxicity of the PD brain homogenates relative to control brain homogenates (Figure 1E). At the highest concentration (1000 µg/mL) both ND and PD samples were overly toxic to the SH-SY5Y cells, and, at the lower concentrations (50, 20 and 0 µg/mL), there was minimal toxicity under the assay conditions employed. However, at total brain protein concentrations of 500, 200 and 100 µg/mL, there was a significant difference in toxicity observed between the PD and control samples (Figure 1E). The differences in toxicity between both the PD-1 and PD-2 and control samples were most pronounced at a protein concentration of 200 µg/mL, so this protein concentration was utilized for further toxicity studies.

**Figure 2:**
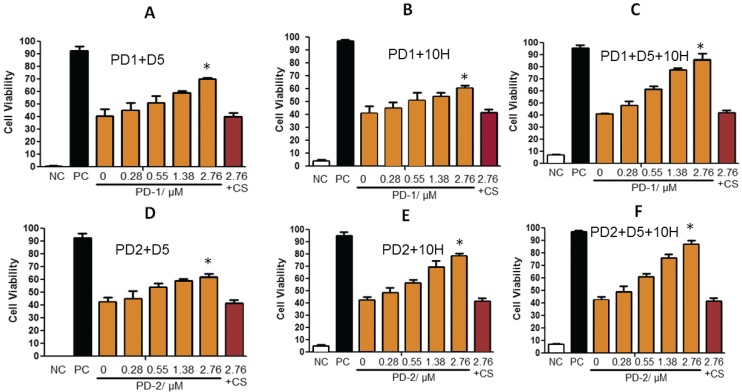
D5 and/or 10H scFv neutralize toxicity of different human PD brain tissue samples (PD-1 and PD-2 mix) toward SH-SY5Y cells. SH-SY5Y cells were treated with PD patient brain homogenized supernatants (200 µg/mL) with or without different concentrations of D5 or/and 10H scFvs (0.28, 0.55, 1.38 and 2.76 µM) for 24 h with medium. NC is the no cell baseline value. PC (Positive control, healthy SH-SY5Y cells treated with control brain tissue, 200 µg/mL). 2.76 + CS is the antibody control group treated with PD patient brain homogenized supernatants (200 µg/mL) and 2.76 µM of a control scFv. Cell viability was determined by the XTT assay. Cell viability is expressed by the mean of three measurements ± 1 standard deviation. Statistical significance was determined between different groups. * is *p* < 0.05.

We next determined if the increased cytotoxicity of the PD brain homogenates was attributable to oligomeric α-synuclein aggregates. When the brain homogenates were added to the SH-SY5Y cells, we also added different concentrations of either D5 or 10H to selectively target and block different α-synuclein aggregates. For the PD-1 brain homogenate sample which contained approximately equivalent amounts of both D5 and 10H reactive oligomeric α-synuclein, cell viability was increased from 40.7% without added scFv to 70.1% at the highest D5 concentration (Figure 2A) and 62.3% at the highest 10H concentration (Figure 2B). When both D5 and 10H scFvs were incubated together with the brain tissue homogenates and cells, the cell viability increased to 91.1% (Figure 2C) neutralizing most of the additional toxicity in the PD-1 sample mix relative to the control brain sample. The PD-2 brain homogenate sample mixture contains brain tissue homogenates that contain higher levels of 10H compared to D5 reactive oligomeric α-synuclein aggregates. When cells were incubated with the PD-2 brain tissue mixture and with 10H scFv, cell viability was increased from 40.7% without added scFv to nearly 80% (Figure 2E), but only to around 60% when co-incubated with D5 (Figure 2D). When both D5 and 10H scFvs were incubated with the PD-2 brain tissue sample the cell viability increased slightly more compared to treatment with 10H alone (87.2%) (Figure 2F).

**Figure 3:**
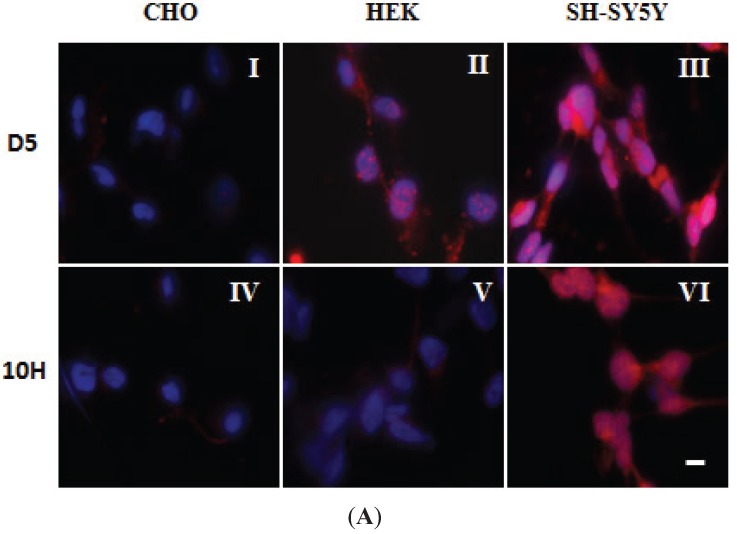

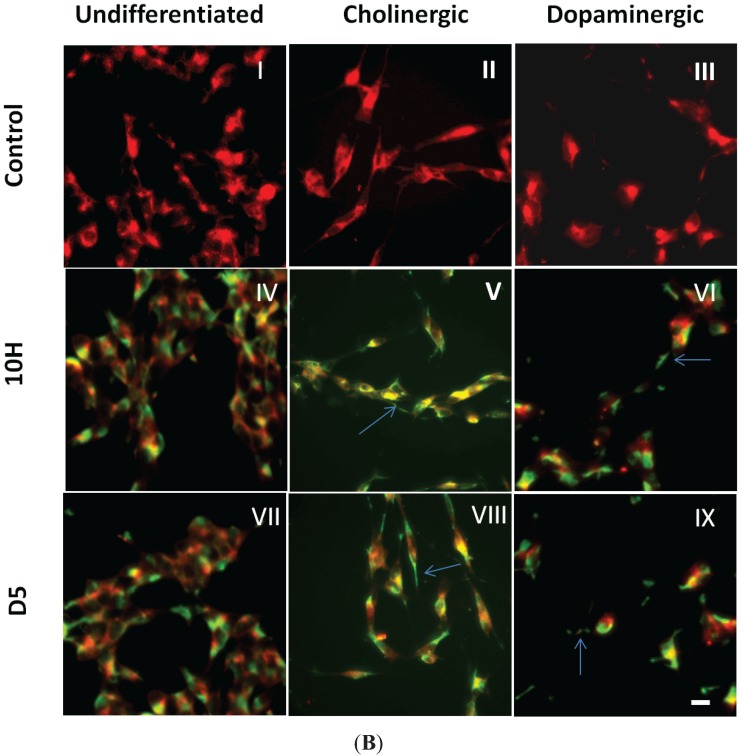
Localization of D5 and 10H reactive α-synuclein aggregates in different cell lines. (**A**) Localization of α-synuclein aggregates in CHO, HEK and SH-SY5Y cells. Cells were fixed with formaldehyde and probed without or with 0.2 mg/mL of D5 (red), 10H (red) and DAPI (blue); (**B**) Staining of D5 and 10H reactive oligomeric α-synuclein morphologies in undifferentiated (I, IV, VII) and differentiated cholinergic-like cell (II, V, VIII) and dopaminergic-like SH-SY5Y cells (III, VI, IX); The undifferentiated cells were treated with anti-synaptophysin (red) (I, IV, VII), the cholinergic-like cells with anti-ChAT (red) (II, V, VIII), and the dopaminergic-like cells with anti-TH, (red) (III, VI, IX). Cells were fixed in 4% formaldehyde and probed without (I, II, III) or with 10H (IV, V, VI) or with D5 (VII, VIII, IX) (green) scFv antibody fragments. Arrows indicate staining in neurites. The scale bar equal to 50 µm.

With both the PD-1 and PD-2 brain mixture samples, addition of both D5 and 10H restored cell viability to around 90% of that observed when cells were incubated with PBS buffer. Therefore, D5 and 10H reactive α-synuclein aggregates are two of the primary pathological toxic α-synuclein species that are present in post-mortem human PD brain tissue. Cell models used to replicate the α-synuclein aggregation process should also be able to replicate the presence of these toxic oligomeric species. To identify which cell models best replicate the α-synuclein pathology observed in the human PD brain, we probed several different mammalian cell lines with D5 and 10H scFvs to determine how well they recreated the α-synuclein aggregation profile found in human brain tissue. We studied how both different cell genotypes (CHO, HEK293 and SH-SY5Y) as well as cell phenotypes (undifferentiated, cholinergic-like and dopaminergic-like SH-SY5Y cells) affect α-synuclein generation *in situ*. The choice of cell line rather dramatically influences which α-synuclein aggregates are generated. CHO cells did not react with either D5 (Figure 3A-I) or 10H (Figure 3A-IV), while HEK cells reacted strongly with D5, but only very weakly with 10H (Figure 3A-II,-V), and SH-SY5Y cells reacted strongly with both 10H (Figure 3A-III) and D5 (Figure 3A-VI).

Because dopaminergic cells are preferentially targeted in PD, we tested whether different neuronal cell phenotypes can alter α-synuclein aggregation. We generated three different neuronal cell phenotypes (non-differentiated, cholinergic-like, and dopaminergic-like) from SH-SY5Y human neuroblastoma cell line by sequential exposure of cells to RA and BDNF using previously described protocols [33,34,35]. These protocols provide homogeneous populations of fully neuronal, differentiated cells that are very comparable to primary neurons and exhibit characteristics of cholinergic and dopaminergic neurons, including expression of choline acetyltransferase (ChAT), vesicular monoamine transporter (VMAT), and tyrosine hydroxylase (TH) [33,34,35,50,51,52,53]. We confirmed cell phenotype by verifying expression of neuronal differentiation markers. Cholinergic-like SH-SY5Y cells treated with RA for five days showed high expression of ChAT (Figure 3B-II), while dopaminergic-like cells treated sequentially with RA and BDNF showed extensive expression of TH (Figure 3B-III). After verifying that we had cholinergic-like and dopaminergic-like neuronal cells, we then probed the different cell types for the presence of oligomeric α-synuclein with D5 and 10H scFvs. The undifferentiated, cholinergic-like and dopaminergic-like SH-SY5Y cells all showed similar labeling with 10H (Figure 3B-IV, -V and -VI), where the 10H reactive α-synuclein aggregates were preferentially localized in the cell body in all cases with only slight staining of neurites. In contrast, the undifferentiated SH-SY5Y cells showed D5 reactive oligomeric α-synuclein aggregates present in the cytoplasm similar to 10H α-synuclein aggregates (Figure 3B-VII), while the cholinergic-like and dopaminergic-like cells also showed significant D5 reactive oligomeric α-synuclein aggregates in the cell neurites and cell body (Figure 3B-VIII,-IX). These results indicate that the D5 reactive α-synuclein species are produced by all three neuronal phenotypes in the cell body, however cholinergic-like and dopaminergic-like cells also produce D5 reactive α-synuclein aggregates in the cell neurites. The 10H reactive oligomeric α-synuclein species occurs predominantly in the cell body for all three neuronal phenotypes, though they are also present in cell neurites in both cholinergic-like and dopaminergic-like cells.

Therefore, a variety of different oligomeric α-synuclein aggregates are produced by different cell types even when α-synuclein is expressed at normal levels. Our results indicate that the type and location of the α-synuclein aggregates generated depend on both cell genotype and phenotype, where dopaminergic and cholinergic neurons preferentially generate a toxic oligomeric α-synuclein species in neurites compared to other cell types. We postulate that in healthy cells these oligomeric aggregates are cleared through normal cellular mechanisms including proteasomal clearance and autophagy [54]; however, when these clearance processes begin to fail, toxic oligomeric α-synuclein species begin to accumulate and may spread to healthy cells through an exosomal mediated mechanism [26,55,56].

**Figure 4:**
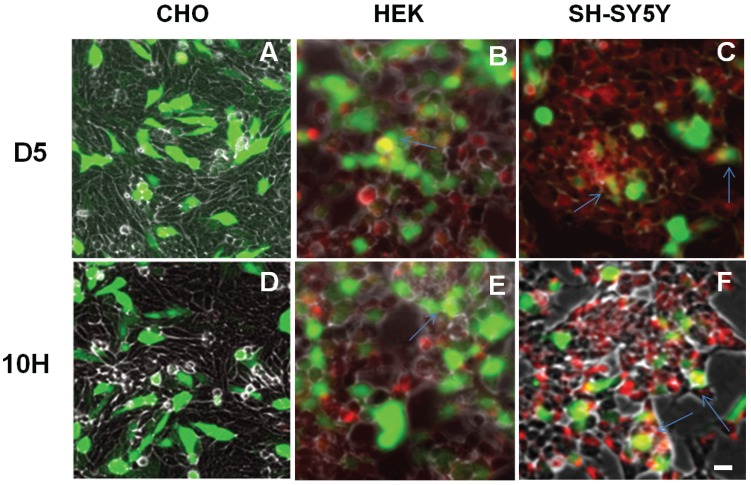
Localization of D5 and 10H reactive α-synuclein aggregates in CHO, HEK and SH-SY5Y cells overexpressing α-synuclein. Cells were seeded in 6-well plates with a density of 10^5^ cells/well and treated with 2:1 transfected reagent and wt-synuclein-EGFP (green). After 72 h, the cells were fixed with formaldehyde and probed without or with D5 or 10H scFv antibody fragments (red). Arrows indicate co-localization. The scale bar equal to 50 µm.

Since overexpression of α-synuclein has been extensively used in cell and animal models of PD, we also studied how overexpression of α-synuclein affects the aggregation state of α-synuclein. We induced α-synuclein overexpression in CHO, HEK, and SH-SY5Y cells by transfection with the WTsynEGFP gene so excess expression of α-synuclein could be readily verified by fluorescence. We then investigated whether there was a change in oligomeric α-synuclein aggregation induced by overexpression. Overexpression of α-synuclein in CHO cells still did not result in the presence of D5 (Figure 4A) or 10H (Figure 4D) reactive α-synuclein aggregates similar to what we observed when the cells expressed endogenous levels of α-synuclein (Figure 3A-I,-IV). Similarly with HEK cells, overexpression of α-synuclein led to the presence of both D5 (Figure 4B) and 10H (Figure 4E) reactive α-synuclein aggregates with much higher presence of D5 reactive aggregates than 10H again similar to results observed with endogenous expression (Figure 3A-II,-V). Finally, with undifferentiated SH-SY5Y cells, 10H and D5 reactive α-synuclein aggregates were observed (Figure 4C,F), again similar to the results observed with endogenous levels of α-synuclein expression (Figure 3A-III,-VI).

**Figure 5:**
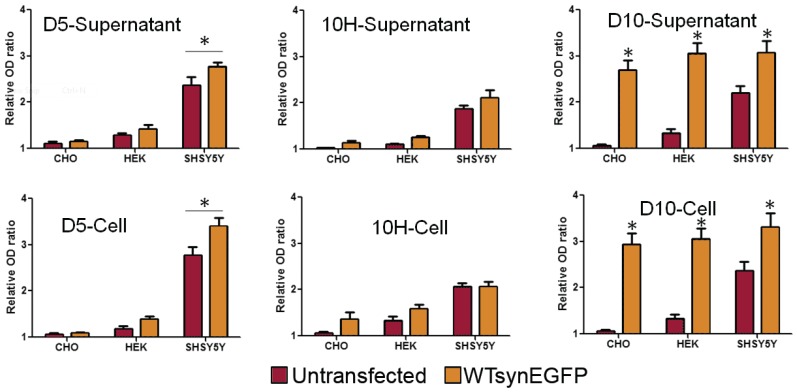
The levels of total α-synuclein (D10) and D5 and 10H reactive oligomeric α-synuclein aggregates in cell supernatant and lysate with endogenous and overexpressed levels of α-synuclein. Levels of the different α-synuclein species were determined by capture ELISA (D5 and 10H) or by indirect ELISA (D10) [49]. Cells (10^5^/mL) were seeded in a six well plate and transfected with or without wt-synuclein-EGFP. α-Synuclein levels are expressed as the mean value of three measurements ± standard deviation. Statistical significance was determined at transfected cells *vs.* normal cells. * is *p* < 0.05.

We quantified the levels of total α-synuclein and the D5, 10H reactive oligomeric α-synuclein in the different cell lines expressing both endogenous and overexpressed levels of α-synuclein by ELISA (Figure 5). Overexpression of α-synuclein in CHO cells did not result in a significant increase in D5 or 10H reactive α-synuclein aggregates in the supernatant or cell lysate although the total α-synuclein levels (D10) were substantially higher in supernatant and lysate. Overexpression of α-synuclein in HEK cells only very modestly increased levels of D5 and 10H reactive oligomeric α-synuclein in both supernatant and lysate cells (*p* < 0.05) (Figure 5). However, overexpression of α-synuclein in undifferentiated SH-SH5Y cells significantly increased levels of D5 reactive oligomeric α-synuclein in both supernatant and cell lysates, while 10H levels reactive α-synuclein levels did not increase substantially with α-synuclein overexpression (Figure 5). Therefore, overexpression of α-synuclein does not induce formation of toxic oligomeric α-synuclein variants in cell lines that do not naturally produce them; however, the levels of different α-synuclein species generated may vary with expression level. These results indicate that in PD models, choice of cell type affects the α-synuclein aggregation process and cytotoxicity much more than expression levels.

In summary, oligomeric α-synuclein aggregates represent the primary cytotoxic α-synuclein species present in post-mortem human PD brain tissue and this toxicity can be largely eliminated by targeting two different morphological species of α-synuclein aggregate using the D5 and 10H scFvs. We further show that when trying to replicate this toxic α-synuclein pathology in PD models, both cell genotype and phenotype strongly influence the aggregation state of α-synuclein, though expression levels do not. Differentiation of SH-SY5Y cells into cholinergic and dopaminergic phenotypes gave distinct α-synuclein aggregation patterns that were similar to those observed with rat primary neurons, suggesting that differentiated neurons may be a reasonable cell model to study α-synuclein toxicity in PD.

## 3. Experimental Section

### 3.1. Materials

#### Cell Culture

*HEK293 Cells*. Human embryonic kidney (HEK293) cells were cultured in Dulbecco’s Modified Eagle Medium (DMEM), from Invitrogen, supplemented with 10% fetal bovine serum (FBS, Sigma, St Louis, MO, USA), 1% penicillin/streptomycin and 2% L-glutamine (Life Technologies, New York, NY, USA) at 37 °C, in a 5% CO_2_ atmosphere. HEK293 cells were generous gifts from Nick Webster (Wadsworth Center, Albany, NY, USA).

*CHO Cells*. Chinese hamster ovary cells (CHO) cells were cultured in the same medium as above, supplemented with 10% fetal bovine serum, 1% penicillin/streptomycin and 1% L-glutamine (Life Technologies) and grown at 37 °C, in a 5% CO_2_ atmosphere. CHO cells [57] were generous gifts from Dennis Selkoe (Harvard Medical School).

*SH-SY5Y Cells*. SH-SY5Y human neuroblastoma cell line cells were maintained in culture flasks in medium containing (*v*/*v*) minimal essential medium (MEM), (*v*/*v*) Ham’s modification of F-12, 10% fetal bovine serum (FBS, Sigma), 1% non-essential amino acid, and 1% antibiotic-antimycotic (Life Technologies) and grown in a 5% CO_2_ atmosphere at 37 °C. The SH-SY5Y cell line was purchased from ATCC (Vienna, VA, USA), minimal essential medium (MEM) and Ham’s modification F-12 were obtained from Irvine Scientific (Santa Ana, CA, USA). Non-essential amino acids and antibiotic-antimycotic 100x were obtained from Invitrogen (Carlsbad, CA, USA). All chemicals were obtained from Sigma-Aldrich (St. Louis, MO, USA) unless otherwise indicated.

*Differentiated SH-SY5Y Cells.* SH-SY5Y cells were harvested from flasks and plated in 6-well polystyrene plates (Corning Inc., Corning, NY, USA) coated previously with 0.5 mg/mL collagen with density of 10^5^ cells per well. The cells were differentiated to cholinergic-like neuronal cells by addition of 10 µM retinoic acid (Sigma-Aldrich) in media with serum for 4 days at 37 °C. After 4 days in the presence of RA, cells were differentiated to dopaminergic-like neuronal cells by incubating with 50 ng/mL of brain-derived neurotrophic factor (BDNF) (PeproTech, Rocky Hill, NJ, USA) in media without serum for 7 days at 37 °C as described [33,34,35,36].

*Transient Transfection of SH-SY5Y, CHO and HEK Cells.* Transient transfection of cells was performed using FuGENE HD Transfection Reagent according to the manufacturer’s protocol (Promega, Madison, WI, USA) with slight modification [13]. Briefly, SH-SY5Y cells (10^5^ cells/well) were seeded in 1 mL of growth medium in 6 well plates for 1 or 2 days before transfection (50%–70% confluence). A transfection mixture consisting of wild type α-synuclein/EGFP (WTsynEGFP) fusion protein plasmid DNA (Clontech, Palo Alto, CA, USA) and Fugene reagent (1:2 *v*/*v*) in serum-free media was pre-incubated in the dark for 15 min. at room temperature before addition to the cells. Cell culture media was removed, and after the transfection mixture (500 μL) was added to each well, the culture was incubated for 1 h at 37 °C, followed by addition of complete media with serum (500 μL). The cultures were incubated and grown in a 5% CO_2_ atmosphere at 37 °C for 72 h. The cells were then probed with D5 or 10H as described above.

### 3.2. Purification of D5, 10H and D10 Fragments and Western Blot

The D5 and 10H single chain antibody fragments (scFv) were purified using a protein A sepharose column as described previously [20,21]. Briefly, the supernatant and periplasmic fractions from a 1 L culture were combined, passed through a 0.2 μm filter (Whatman, Clifton, NJ, USA), and then concentrated in a tangential flow filter (Millipore, Billerica, MA, USA) using a 10 kDa filter membrane (Millipore). Concentrated samples were applied to a protein A-Sepharose column (GE healthcare, NJ, USA) which was equilibrated in PBS 1x, pH 7.4, at 4 °C. After washing the column in the same buffer, bound antibody fragments were eluted from the column with 0.2 M glycine, pH 3. Fractions containing antibody fragments were pooled, adjusted to neutral pH, dialyzed into PBS and stored at −20 °C. The purity of the antibody fragments was estimated by electrophoresis on 12% (*w*/*v*) SDS-polyacrylamide gels and Western Blot (method see Emadi *et al*., 2007) [20,21].

### 3.3. Preparation of Brain Tissue Homogenates

Post-mortem brain tissue samples from pathologically verified age-matched non-diseased (ND) and Parkinson’s disease (PD) patients were generously provided by Dr. Thomas Beach (Civin Laboratory for Neuropathology, Sun Health Research Institute, Sun City, AZ, USA) (Table 1). The middle temporal gyrus (MTG) of ten different PD samples and five age matched cognitively normal ND samples were separately homogenized in 0.1 M Tris/Lysis buffer (pH 7.4) with protease inhibitor (Pierce), centrifuged and combined as PD and ND samples, respectively. The PD samples (Table 1) were split into two groups of five samples each based on the relative amounts of D5 and 10H reactive oligomeric α-synuclein aggregates present in each sample where PD-1 group contained samples with relatively similar amounts of D5 and 10H reactive α-synuclein aggregates and PD-2 group contained samples with predominantly 10H reactive α-synuclein aggregates [49].

**Table 1:** Human Brain Samples for Parkinson’s Disease PD-1, PD-2 and ND.

**Sample**	**Gender**	**Age**
**PD-1**	M	85
	F	73
	M	75
	F	74
	M	77
**PD-2**	F	78
	M	72
	F	91
	F	82
	M	70
**ND**	M	79
	M	65
	F	87
	M	89
	F	83

### 3.4. Incubation of Human Brain Tissue Homogenates with SH-SY5Y Cells

The toxicity of the brain tissue samples towards human neuroblastoma SH-SY5Y cells was measured using an XTT assay [48]. SH-SY5Y cell line were cultured at 37 °C in a humidified atmosphere with 5% CO_2_, in a HEPES (20 mM)-buffered RPMI 1640 cell culture medium supplemented with 2 mM L-glutamine, antibiotic/antimycotic mixture (1%) and 10% fetal bovine serum (FBS). Cells were treated with PD patient brain homogenized supernatants (100 µg/mL) with or without added D5 or 10H scFv (0, 0.28, 0.55, 1.38 and 2.76 μM of scFv in final medium) for 24 h with above medium. ND brain homogenized supernatants was used as control sample to show the toxicity background. Cell viability was determined by the XTT assay. The cells incubated in 96-well flat-bottom plates for the cell viability assessment were 2 × 10^4^ cells/well. A freshly prepared XTT-PMS labeling mixture (50 μL) was added to the cell culture and incubated for 3–4 h. The absorbance was measured at 450 nm and 570 nm in an automated microplate reader. All sample values were normalized to a mixture containing five control patient samples (ND).

### 3.5. Immunofluorescent Staining

Cells were fixed in 4% formaldehyde in PBS at room temperature for 15 min. Then cells were treated with 0.2% Triton 100 at room temperature for 15 min., washed three times with PBS and blocked for 1 h with PBS containing 5% goat serum. The cells were incubated overnight (ON) with 0.3 mg/mL of D5 and/or 10H scFv. The primary antibodies (mouse anti-c-Myc (Sigma), anti-Synaptophysin (Santa Cruz Biotechnology, Dallas, TX, USA), anti-choline acetyltransferase (Santa Cruz Biotechnology) and anti-tyrosine hydroxylase, all at a 1/100 dilution) were then applied to the cells, which were incubated for 2 h at room temperature. The cells were washed 3 times with PBS and incubated with 1/1000 dilution of the secondary antibodies (goat anti-mouse IgG Alexa Fluor 488 and goat anti-rabbit Alexa Fluor 594, Life Technologies) or DAPI (Sigma) for 1 h at room temperature. The images were taken with a fluorescent microscope (Observer D1, Zeiss).

### 3.6. Capture ELISA

High binding polystyrene microtiter plates were coated with 0.3 µg/mL of D5 or 10H scFv in carbonate-bicarbonate, pH 9.6 at 4 °C for overnight. Non-specific binding was blocked by incubating with 2% PBS-milk at 37 °C for 1 h. The plates were then incubated with an aliquot of supernatant or cell lysate from untransfected and transfected SHSY5Y, CHO and HEK cells, for 2 h at 37 °C. After washing, an aliquot of 10^10^ titer units of PEG precipitated D10 phage in 100 µL of 2% PBS-milk was added to each well and incubated for 90 min at room temperature. Bound phages were detected after 1 h incubation with a 1:2000 dilution of avidin- horseradish peroxidase (HRP) conjugate. A 100 µL aliquot of the HRP substrate 3.3'.5.5'-tetramethylbenzidine (TMB, Sigma) was added and the reaction was stopped after 20 min with 2 M H_2_SO_4_. The activity was determined by subtracting OD_650_ from OD_450_ using a Wallac 1420 plate reader (Perkin Elmer, Waltham, MA, USA) and comparing absorbance between control well (without D10 phage) to the sample well (with D10 phage) where the control value was normalized to 1.

To measure oligomer content in the brain tissue the wells were coated with 0.3 µg /mL of 10H or D5 scFv for 1 h at 37 °C. The wells were then blocked for 1 h at 37 °C with 2% milk. Brain tissue was added at a concentration of 100 ug/mL for 1 h at 37 °C followed by the detection antibody, biotinylated D10 phage, at 1/1000 dilution. Next, a 1/1000 dilution of avidin-HRP (Sigma-Aldrich) was added to wells and binding detected using the SuperSignal ELISA Femto Maximum Sensitivity Substrate kit (Thermo Scientific, Waltham, MA, USA). Signals were measured using a Wallac Victor^2^ microplate reader after 1 and 20 min. Signals were compared to the PBS controls (PBS added in place of brain tissue) and then to the NDs.

### 3.7. Statistical Analysis

All the experiments were performed in triplicate unless otherwise stated. The data in the text and figures are expressed as mean ± 1 SEM. Statistical comparisons between groups were assayed using *t* test. One way ANOVA was used to test for significance. *P*-values less than 0.05 were considered significant. In the ELISAs, the PDs were normalized relative to the NDs. All the analyses were performed using SPSS for Windows version 13.0 (SPSS Software, IBM, Hong Kong, China).

## 4. Conclusions

In summary, oligomeric α-synuclein aggregates represent the primary cytotoxic α-synuclein species present in post-mortem human PD brain tissue and this toxicity can be largely eliminated by targeting two different morphological species of α-synuclein aggregate using the D5 and 10H scFvs. We further show that, when trying to replicate this toxic α-synuclein pathology in PD models, both cell genotype and phenotype strongly influence the aggregation state of α-synuclein, though expression levels do not. Differentiation of SH-SY5Y cells into cholinergic and dopaminergic phenotypes gave distinct α-synuclein aggregation patterns that were similar to those observed with rat primary neurons, suggesting that differentiated neurons may be a reasonable cell model to study α-synuclein toxicity in PD.

## Abbreviations

PD: Parkinson’s disease
PD: Parkinson’s disease
scFv: single chain antibody fragment
AFM: atomic force microscope
A30P: human A30P α-synuclein mutation
A53T: human A53T α-synuclein mutation
E46K: human E46K α-synuclein mutation
SH-SY5Y: human neuroblastoma cells
CHO: Chinese hamster ovary cells
HEK: human embryonic kidney cells
TH: tyrosine hydroxylase
RA: retinoic acid
BDNF: brain derived neurotrophic factor
ChAT: choline acetyltransferase
WTsynEGFP: α-synuclein/EGFP fusion protein
